# Therapeutic effectiveness of percutaneous drainage and factors for performing an interval appendectomy in pediatric appendiceal abscess

**DOI:** 10.1186/s12893-016-0188-4

**Published:** 2016-10-18

**Authors:** Chih-Cheng Luo, Kuang-Fu Cheng, Chen-Sheng Huang, Hung-Chieh Lo, Sheng-Mao Wu, Hung-Chang Huang, Wen-Kuei Chien, Ray-Jade Chen

**Affiliations:** 1 Division of Pediatric Surgery, Department of Surgery, Wan Fang Medical Center, Taipei Medical University, 111 Xinglong Rd., Sect. 3, 11696 Taipei, Taiwan; 2 Department of Traumatology, Wan Fang Medical Center, Taipei Medical University, Taipei, Taiwan; 3 Department of Acute Care Surgery and Traumatology, Taipei Medical University, Taipei, Taiwan; 4 Department of Surgery, Taipei Medical University Hospital, Taipei Medical University, 252 Wu-Xing Street, Taipei, 110 Taiwan; 5 Biostatistics Center, Taipei Medical University, Taipei, Taiwan; 6 Division of Pediatric Surgery, Department of Surgery, School of Medicine, College of Medicine, Taipei Medical University, No.250, Wuxing St, Taipei, 11031 Taiwan

**Keywords:** Appendiceal abscess, Percutaneous drainage, Interval appendectomy

## Abstract

**Background:**

In this study, we studied the therapeutic effectiveness of percutaneous drainage with antibiotics and the need for an interval appendectomy for treating appendiceal abscess in children with a research-oriented dataset released by the Bureau of National Health Insurance in Taiwan through the Collaboration Center for Health Information Application (CCHIA).

**Methods:**

We identified 1225 patients under 18 years of age who had non-surgical treatment for an appendiceal abscess between 2007 and 2012 in a Taiwan CCHIA dataset. The treatment included percutaneous drainage with antibiotics or antibiotics alone. We also analyzed data of patient’s baseline characteristics, outcomes of percutaneous drainage, and indicating factors for performing an interval appendectomy.

**Results:**

Totally, 6190 children had an appendiceal abscess, an 1225 patients received non-operative treatment. Of 1225 patients, 150 patients received treatment with percutaneous drainage and antibiotics, 78 had recurrent appendicitis, 185 went on to receive an interval appendectomy, and 10 had postoperative complications after the interval appendectomy. We found that patients treated with percutaneous drainage and antibiotics had a significantly lower rate of recurrent appendicitis (*p* < 0.05), a significantly smaller chance of receiving an interval appendectomy (*p* < 0.05), and significantly fewer postoperative complications after the interval appendectomy (*p* < 0.05) than those without percutaneous drainage treatment. Older children (13 ~ 18 years) patients were found to have a significantly smaller need to receive an interval appendectomy than those who were ≤ 6 years of age (odd ratio (OR) = 2.071, 95 % confidence interval (CI) = 1.34–3.19, *p <* 0.01), and those who were 7 ~ 12 years old (OR = 1.662, 95 % CI = 1.15–2.41, *p <* 0.01). In addition, those treated with percutaneous drainage were significantly less indicated to receive an interval appendectomy later (OR = 2.249, 95 % CI = 1.19 ~ 4.26, *p <* 0.05). In addition, those with recurrent appendicitis had a significantly increased incidence of receiving an interval appendectomy later (OR = 3.231, 95 % CI = 1.95 ~ 5.35, *p* < 0.001).

**Conclusions:**

In this study, we used nationwide data to demonstrate therapeutic effectiveness of percutaneous drainage and antibiotics was more beneficial than only antibiotics in treating patients with an appendiceal abscess. We also found three factors that were significantly associated with receiving an interval appendectomy: recurrent appendicitis, being aged ≤ 13 years, and treatment with antibiotics only.

## Background

Appendicitis is a common pediatric surgical emergency. Even with the use of ultrasonagraphy and computed tomography (CT), perforation rates in children have been reported to be 30 % over the last 30 years, and some perforated patients presented themselves with an appendiceal abscess [1–3].

Appendiceal abscess can immediately be treated by surgery or by non-surgical management, consisting of treatment with parenteral antibiotics alone, or treatment with antibiotics and ultrasound- or CT-guided drainage, followed by an interval appendectomy [4, 5]. But the need for a future interval appendectomy remains debatable [6–10]. Without an interval appendectomy, the risks of recurrent appendicitis and missed pathological findings are uncertain. The recurrence rate after treatment with percutaneous drainage and antibiotics for patients with an appendiceal abscess was found to be low, and an interval appendectomy in most cases was not required [11, 12].

In this study, we used dataset from Taiwan’s National Health Insurance (NHI) to analyze the effectiveness of percutaneous drainage therapy, and to determine the possible indicating factors for performing an interval appendectomy.

## Methods

### Data source

This study used data from the Taiwan National Health Insurance (NHI) program, which the government initiated in 1995, and which covers 99 % of the population of 23 million people. The Bureau of the NHI (BNHI) in Taiwan has released a research-oriented database through the Collaboration Center for Health Information Application (CCHIA). In 1999, the BNHI began to release all claims data in electronic form, to allow researchers to trace almost all uses of medical services for all people in Taiwan, including all children with appendicitis.

We extracted datasets between 2007 and 2012 from the NHI research database (NHIRD) released by the BNHI through the CCHIA. The study protocol was approved by the Taipei Medical University Joint Institutional Review Board without the need to obtain signatures of study participants, because the NHIRD consists of de-linked secondary data.

### Study sample

We enrolled 1225 pediatric patients (≤18 years of age) with a diagnosis of an appendiceal abscess who received non-surgical treatment between January 2007 and December 2012, because the *International Classification of Disease, Ninth Revision, Clinical Modification (ICD-9-CM)* codes were available since 2007.

Inpatient claims, which included records of all hospitalizations, provided a substantial amount of information. We linked study participants to the inpatient claims data to identify appendiceal abscess (540.9), percutaneous drainage (470.22C) and postoperative complications include an intra-abdominal abscess (IAA,998.59) and postoperative bowel obstruction (PBO,560.81 or 997.4) based on ICD-9-CM codes.

Patients were divided into three age groups: ≤ 6, 7 ~ 12, and 13 ~ 18 years old. The definition of an interval appendectomy is a patient who had initial medical therapy with intravenous antibiotics or possibly drainage followed by an elective appendectomy. The definition of recurrent appendicitis is a patient who has appendicitis again after initial non-surgical treatment without an appendectomy. Excluded from the study were patients for whom the initial non-surgical treatment failed or who had recurrent appendicitis and received immediate surgery.

### Statistical analysis

We performed a univariate analysis between different age group and treatment methods. We present continuous variables as the mean ± standard deviation (SD) and analyzed differences in these parametric variables between groups with Student’s *t*-test. We also present categorical variables as ratios, and analyzed differences in non-parametric variables between groups with Chi-square test. A multivariate logistic regression analysis was used to identify independent factors including factors of different age groups, gender, the use of antibiotics, the use of percutaneous drainage, and recurrent or non-recurrent appendicitis. The relationship between potential variables and the necessity for an interval appendectomy was assessed by odds ratios (ORs) and related 95 % confidence intervals (CIs).

We used the Statistical Analytic System software version 9.3 (SAS Institute, Cary, NC, USA) to analyze the data in this study. Differences between groups were considered significant if *p* values were < 0.05.

## Results

Totally, 6190 children had an appendiceal abscess, and non-surgical treatment was used in 1,225 patients. Table 1 lists of demographic data of all patients with an appendiceal abscess. Of 1225 patients, 185 received an interval appendectomy, 78 had recurrent appendicitis, and 10 had postoperative complications after an interval appendectomy.

**Table 1:** Demographic data of appendiceal abscess patients

Variable	Patients with aappendiceal abscess	Non-operative treatment	Drainage	Recurrence	Interval appendectomy
		*n* (%)	*n* (%)	*n* (%)	*n* (%)
Age group (years)					
≤ 6	628	233 (37.1)	27 (11.6)	18 (7.8)	47 (20.2)
7 ~ 12	2296	494 (21.5)	55 (11.1)	29 (5.8)	84 (17.0)
13 ~ 18	3266	498 (15.2)	68 (13.7)	31 (6.2)	54 (10.8)
Gender					
Male	3786	687 (18.15)	90 (13.1)	42 (6.1)	98 (14.3)
Female	2404	538 (22.38)	60 (11.2)	36 (6.6)	87 (16.1)
Total	6190	1225 (19.8)	150 (12.2^a^)	78 (6.3^a^)	185 (15.1^a^)

^a^ Percentage who received non-operative treatment

Of the 1225 children presenting with an appendiceal abscess had non-surgical treatment. 150 (2.2 %) patients were treated by percutaneous drainage with antibiotics, and the remaining 1075 (97.8 %) were only given antibiotics without percutaneous drainage. The average time of hospital stay for patients treated with percutaneous drainage (at 12.8 ± 8.9 days) was longer than that without drainage (9.7 ± 6.4 days) (*p* < 0.05). Table 2 demonstrates that patients treated with percutaneous drainage and antibiotics had significantly lower rates of recurrent appendicitis (*p* < 0.05), a significantly smaller chance of receiving an interval appendectomy (*p* < 0.05), and a significantly smaller chance of postoperative complications (*p* < 0.05) after an interval appendectomy than those without percutaneous drainage treatment.

**Table 2:** Comparison of therapeutic effectiveness in patients with and without percutaneous drainage

	Without drainage (%)	With drainage (%)
R ecurrence		
Yes	73 (6.79)	5 (3.33) ^a^
No	1002 (93.21)	145 (96.67)
Interval appendectomy (IA)		
Yes	174 (16.19)	11 (7.33) ^a^
No	901 (83.81)	139 (92.67)
Postoperative complications after IA		
Yes	10 (5.75)	0 (0) ^a^
No	164 (94.25)	11 (100)

^a^ Significantly greater therapeutic effectiveness in patients with drainage compared with those without drainage (*p* < 0.05)

In our study (Table 1), 185 (15.1 %) patients received an interval appendectomy and the remaining 1040 (84.9 %) did not. Data from the multivariate logistic regression analyses (Table 3) showed that pediatric patients who received percutaneous drainage had a significantly smaller need to receive an interval appendectomy (OR = 2.249, 95 % CI = 1.187 ~ 4.260, *p* < 0.05), older (13 ~ 18 years) patients had a significantly smaller need to receive an interval appendectomy than those who were ≤ 6 years, (OR = 2.071, 95 % CI = 1.34 ~ 3.19, *p <* 0.01), and those who were 7–12 years old (OR = 1.662, 95 % CI = 1.15 ~ 2.41, *p <* 0.01), and patients with recurrent appendicitis were significantly likely to have an increased incidence of receiving an interval appendectomy (OR = 3.231, 95 % CI = 1.95 ~ 5.35, *p* < 0.001).

**Table 3:** Multivariate logistic regression of indicating factors for performing an interval appendectomy

Variable	Odds ratio	95 % Confidence interval	
Gender			
Female	1	-	-
Male	1.025	0.743–1.414	
Age group (years)			
13–18	1	-	-
7–12**	1.662	1.146–2.410	
≤ 6**	2.071	1.344–3.193	
Drainage			
Yes	1	-	-
No*	2.249	1.187–4.260	
Recurrence			
No	1	-	-
Yes***	3.231	1.950–5.354	

*p < 0.05, **p < 0.01, ***p < 0.001

## Discussion

To the best of our knowledge, this is the first study to use a large nationwide database to study the effectiveness of percutaneous drainage therapy, and to determine the indicating factors for receiving an interval appendectomy in patients with appendiceal abscess.

Some recent reports mentioned that treatment of acute appendicitis with abscess formation or phlegmon with non-surgical management in children followed by an elective appendectomy has become well-recognized and accepted [13–15]. Non-surgical treatment of a pediatric appendiceal abscess is now also an acceptable approach in Taiwan. As shown in Table 1, 19.8 % of patients with appendiceal abscess received non-surgical treatment, consisting of parenteral antibiotics alone and ultrasound- or CT-guided drainage, followed by an interval appendectomy [4, 5]. We found that 12.2 % of patients received treatment with percutaneous drainage and antibiotics, but another 87.8 % of them received only antibiotics. Older children (13 ~ 18 years of age) had an increased incidence of receiving percutaneous drainage in our study. In our opinion, the reason may have been that the technique of drainage is more easily to be performed in older children who are physically larger.

Previous reports revealed that percutaneous drainage with the use of antibiotics is more efficient than treatment with antibiotics alone to successfully and completely treat appendiceal abscess without an interval appendectomy [11, 12]. We had the same findings (Table 2), showing that patients treated with percutaneous drainage and antibiotics had a significantly not only decreased rate of receiving an interval appendectomy (*p* < 0.05), but also had a significantly lower rate of recurrent appendicitis (*p* < 0.05) and had a significantly smaller chance of having postoperative complications (*p* < 0.05) after an interval appendectomy. The average time of hospital stay (12.8 ± 8.9 days) was longer for patients treated with percutaneous drainage than those without drainage (9.7 ± 6.4 days) but with antibiotics alone (*p* < 0.05). Based on these data, we suggest that the therapeutic effectiveness of percutaneous drainage may be due to patients’ hospitalization until drainage was completely stopped.

There are few reports dealing with indicating factors for performing an interval appendectomy. The presence of an appendicolith, increased blood C-reactive protein levels, elevated percent bands of white blood cells, and partial small bowel obstruction on admission are reported to be associated with an increased risk of recurrent appendicitis, and thus, such patients should receive an interval appendectomy [16–18]. In the multivariate logistic regression analyses from our national database (Table 3), we found that pediatric patients had a significantly smaller need to receive an interval appendectomy later on if they had received percutaneous drainage (OR = 2.249, *p* < 0.05) and were significantly older (13 ~ 18 years) *(p* < 0.01). But if the pediatric patients had recurrent appendicitis, they had a significantly greater need to receive an interval appendectomy (OR = 3.231, *p* < 0.001).

### *S*tudy limitations

Readers are warned against over-interpreting our study data because it has two major limitations. First, with a population-based database, we had no other available clinical data including patients’ descriptions of clinical presentation, laboratory data, severity of appendiceal abscess, or pathologic confirmation of an appendiceal abscess. Second, we were unsure whether the staff surgeon decided to treat patients with non-surgical treatment, the use of percutaneous drainage, and performing an interval appendectomy.

## Conclusions

In this our study, we used nationwide data in Taiwan to demonstrate that the therapeutic effectiveness of percutaneous drainage and antibiotics was more beneficial than antibiotics alone in treating patients with appendiceal abscess. We also found three factors significantly associated with receiving an interval appendectomy: recurrent appendicitis, being aged less than 13 years, and treatment with antibiotics only.

## Abbreviations

BNHI: Bureau of NHI
CCHIA: Collaboration Center for Health Information Application
NHI: National Health Insurance
